# Biosynthesis of phlorisovalerophenone and 4-hydroxy-6-isobutyl-2-pyrone in *Escherichia coli* from glucose

**DOI:** 10.1186/s12934-016-0549-9

**Published:** 2016-08-30

**Authors:** Wei Zhou, Yibin Zhuang, Yanfen Bai, Huiping Bi, Tao Liu, Yanhe Ma

**Affiliations:** 1 Tianjin Institute of Industrial Biotechnology, Chinese Academy of Sciences, Tianjin, 300308 China; 2 Key Laboratory of Systems Microbial Biotechnology, Chinese Academy of Sciences, Tianjin, 300308 China; 3 University of Chinese Academy of Sciences, Beijing, China

**Keywords:** Isovaleryl-CoA, Phlorisovalerophenone, 4-Hydroxy-6-isobutyl-2-pyrone, Valerophenone synthase, *Escherichia coli*

## Abstract

**Background:**

Type III polyketide synthases (PKSs) contribute to the synthesis of many economically important natural products, which are typically produced by direct extraction from plants or synthesized chemically. For example, humulone and lupulone (Fig. 1a) in hops (*Humulus lupulus*) account for the characteristic bitter taste of beer and display multiple pharmacological effects. 4-Hydroxy-6-methyl-2-pyrone is a precursor of parasorboside contributing to insect and disease resistance of plant *Gerbera hybrida*, and was recently demonstrated to be a potential platform chemical.

**Fig. 1:**
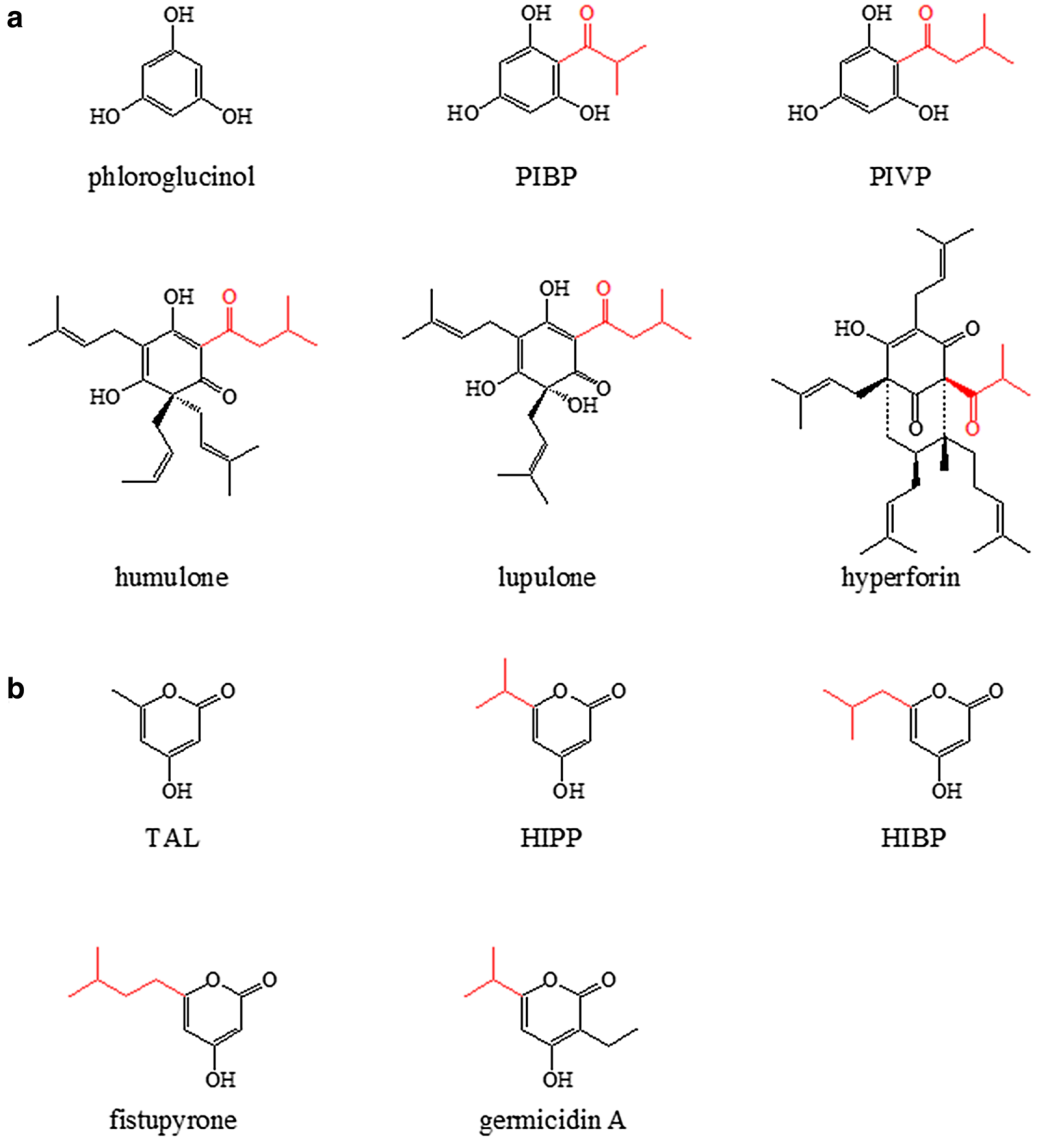
Examples of phloroglucinols (**a**) and 2-pyrones (**b**) synthesized by type III PKS. *PIBP* phlorisobutyrophenone; *PIVP* phlorisovalerophenone; *TAL* 4-hydroxy-6-methyl-2-pyrone (triacetic acid lactone); *HIPP* 4-hydroxy-6-isopropyl-2-pyrone; *HIBP* 4-hydroxy-6-isobutyl-2-pyrone

**Results:**

In this study, we achieved simultaneous biosynthesis of phlorisovalerophenone, a key intermediate of humulone biosynthesis and 4-hydroxy-6-isobutyl-2-pyrone in *Escherichia coli* from glucose. First, we constructed a biosynthetic pathway of isovaleryl-CoA via hydroxy-3-methylglutaryl CoA followed by dehydration, decarboxylation and reduction in *E. coli*. Subsequently, the type III PKSs valerophenone synthase or chalcone synthase from plants were introduced into the above *E. coli* strain, to produce phlorisovalerophenone and 4-hydroxy-6-isobutyl-2-pyrone at the highest titers of 6.4 or 66.5 mg/L, respectively.

**Conclusions:**

The report of biosynthesis of phlorisovalerophenone and 4-hydroxy-6-isobutyl-2-pyrone in *E. coli* adds a new example to the list of valuable compounds synthesized in *E. coli* from renewable carbon resources by type III PKSs.

**Electronic supplementary material:**

The online version of this article (doi:10.1186/s12934-016-0549-9) contains supplementary material, which is available to authorized users.

## Background

A large number of natural products are synthesized by type III polyketide synthases (PKSs). These compounds play an important role in human nutrition and health, and have recently expanded their roles as platform chemicals. Acylphloroglucinol derivatives have been isolated from a number of plants [1–3]. Their chemical structures and intriguing biological activities have attracted increasing attention in recent years. Humulone and lupulone (Fig. 1a) in hops (*Humulus lupulus*) account for the characteristic bitter taste of beer and display multiple pharmacological effects [4]. Hyperforin (Fig. 1a) is one of the main active constituents in extracts of *Hypericum perforatum* used for the treatment of depression [5]. The 4-hydroxy-2-pyrone analogues display multiple biological activities and are valuable pharmaceutical precursors [6]. 4-Hydroxy-6-methyl-2-pyrone, also known as triacetic acid lactone (TAL, Fig. 1b) is a precursor of gerberin and parasorboside, which could protect *Gerbera hybrid* from fungal pathogens and attack by insects [7]. Fistupyrone (4-hydroxy-6-isovaleryl-2-pyrone) (Fig. 1b) was isolated from *Streptomyces* sp. TP-A0569 and showed inhibition of the infection of Chinese cabbage by a fungus *Alternaria brassicicola* [8]. Germicidin homologs (Fig. 1b) arisen from streptomyces inhibit spore germination and Na^+^/K^+^ ATPase [9, 10]. More recently, 4-hydroxy-2-pyrones were reported to be potential platform chemicals (Additional file 1: Figure S1a) [11–13].

Advances in synthetic biology and metabolic engineering greatly promoted biosynthesis of a variety of valuable compounds, for instance, flavonoids produced by type III PKSs [14, 15] in microbial hosts. Biological production of phloroglucinol (Fig. 1a) and TAL (Fig. 1b) from glucose has also been accomplished using genetically modified microorganisms [6, 16–19]. However, biosynthesis of acylphloroglucinols and TAL analogues from renewable feedstocks in *Escherichia coli* has not been well investigated.

The acylphloroglucinol cores, phlorisovalerophenone (PIVP) and phlorisobutyrophenone (PIBP) are formed by condensation of three malonyl-CoA-derived acetate units with isovaleryl-CoA or isobutyryl-CoA as the starter units [4, 20]. The enzyme valerophenone synthase (VPS) involved in the formation of PIVP in the biosynthesis of humulone has been characterized [21–23]. VPS is a homologue of chalcone synthase (CHS). They share the same reaction mechanism namely claisen condensation, but differ in substrate specificity. CHS catalyzes condensation of one molecule of *p*-coumaroyl-CoA and three molecules of malonyl-CoA to form naringenin-chalcone. However, VPS preferentially uses isovaleryl-CoA or isobutyryl-CoA instead of *p*-coumaroyl-CoA as the starter unit to condense with three molecules of malonyl-CoA to form PIVP [21, 23]. Recently, dual functional CHS/VPS have also been reported. A CHS, FvCHS2-1 from strawberry (*Fragaria vesca*) was identified to be responsible for acylphloroglucinols synthesis in strawberry fruit [3]. Intriguingly, in vitro experiments demonstrated that type III PKSs such as VPS and CHS usually also synthesize TAL analogues, 4-hydroxy-6-isobutyl-2-pyrone (HIBP) and 4-hydroxy-6-isopropyl-2-pyrone (HIPP) (Fig. 1b), carrying isobutyl or isopropyl at position 6 with isovaleryl-CoA or isobutyl-CoA as the starter unit and malonyl-CoA as the extender unit.

**Fig. 2:**
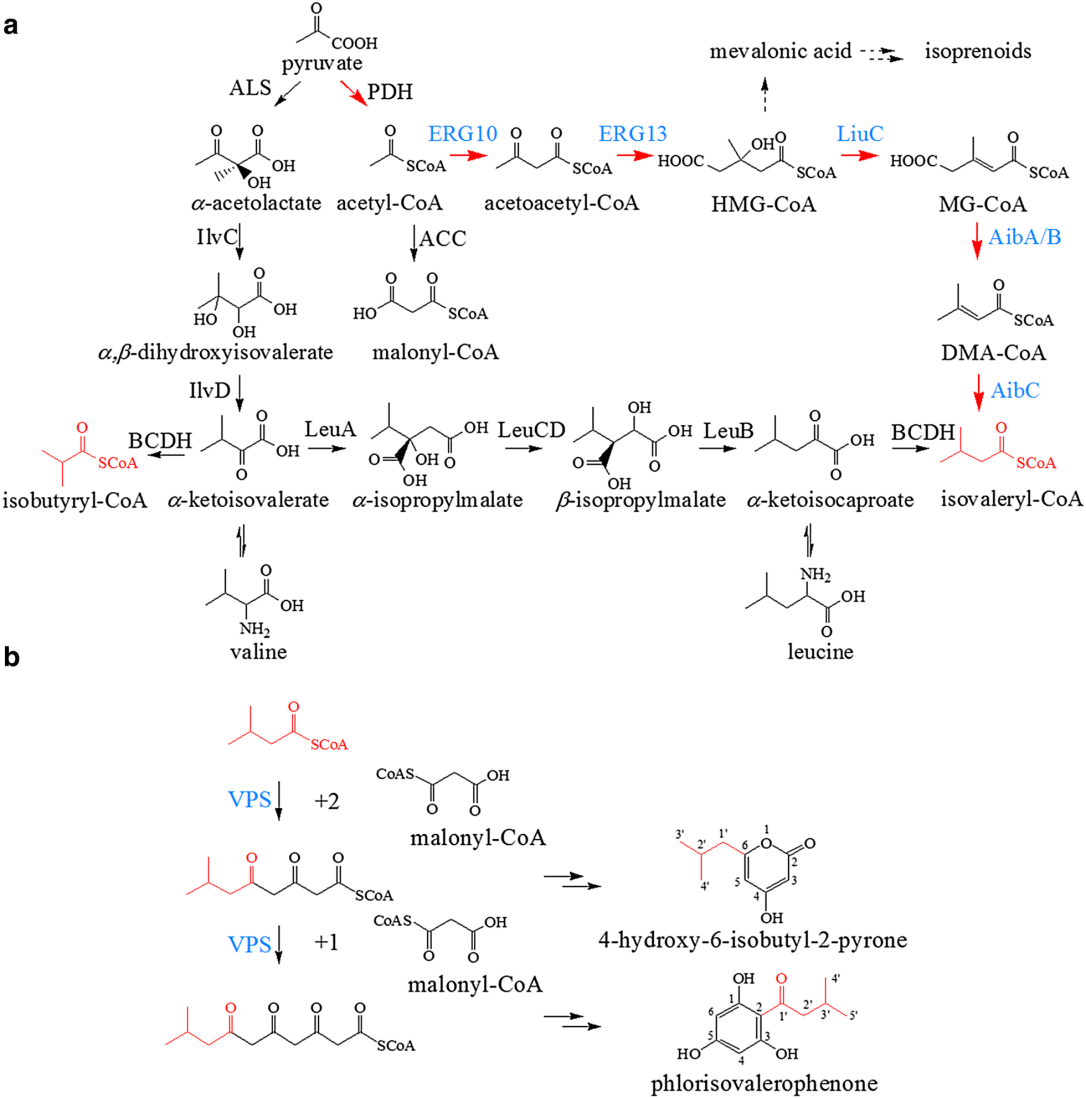
The biosynthetic pathway of phlorisovalerophenone (PIVP) and 4-hydroxy-6-isobutyl-2-pyrone (HIBP) constructed from glucose in *E. coli*. **a** The biosynthetic pathway of isovaleryl-CoA. *Red arrows* represent the constructed isovaleryl-CoA biosynthetic pathway via HMG-CoA followed by dehydration, decarboxylation and reduction. *Black arrows* represent the biosynthesis of isovaleryl-CoA catalyzed by the BCDH complex. *Dotted arrows* represent the mevalonate-dependent pathway of isoprenoid. **b** The formation of PIVP and HIBP catalyzed by VPS. *PDH* pyruvate dehydrogenase complex, *ACC* acetyl-CoA carboxylase, *ERG10* acetyl-CoA acetyltransferase, *ERG13* HMG-CoA synthase, *LiuC* HMG-CoA dehydratase, *AibA/B* MG-CoA decarboxylase, *AibC* DMA-CoA reductase, *Als* acetolactate synthase, *IlvC* ketol-acid reductoisomerase, *IlvD* dihydroxy-acid dehydratase, *LeuA* α-isopropylmalate synthase, *LeuCD* α-isopropylmalate isomerase complex, *LeuB* isopropylmalate dehydrogenase, *BCDH* branched-chain α-keto acid dehydrogenase complex, *BCAT* branched-chain amino acid transaminase, *VPS* valerophenone synthase

In this work, we engineered the synthesis of isovaleryl-CoA in *E. coli* by recruiting a biosynthetic pathway via hydroxy-3-methylglutaryl CoA (HMG-CoA), an intermediate of the mevalonate pathway (Fig. 2a) [24]. The VPS from hops (*Humulus lupulus*) HlVPS, the CHS from strawberry (*Fragaria vesca*) FvCHS2-1 and a newly cloned CHS from *H. perforatum* HpCHS were introduced into the isovaleryl-CoA producing *E. coli* strain separately, led to the simultaneous production of PIVP and HIBP at different ratios. The highest titers of PIVP and HIBP produced by the recombinant strains reached 6.4 and 66.5 mg/L, respectively. This work adds new products to the list of valuable compounds biosynthesized in *E. coli* using type III PKSs and lays a foundation for microbial synthesis of not only acylphloroglucinol derivatives, but also potential platform chemicals.

## Methods

### Strains, plasmids and medium

Strains and plasmids used in this study are listed in Table 1. Luria–Bertani (LB) medium was used for the propagation of *E. coli* cells for plasmid construction. Terrific Broth (TB) medium (12 g/L tryptone, 24 g/L yeast extract, 4 mL/L glycerol, 2.3 g/L KH_2_PO_4_, and 12.5 g/L K_2_HPO_4_) and modified M9 medium (1 × M9 minimal salts, 20 g/L glucose, 10 g/L yeast extract, 2 mM MgSO_4_, and 0.1 mM CaCl_2_) were used for protein expression and PIVP production, respectively. Ampicillin (100 mg/L) and streptomycin (100 mg/L) were added to the medium as needed.

**Table 1:** Bacterial strains and plasmids

Name	Description	Reference
Plasmids		
pETDuet-1	pBR322 ori with P_T7_; Amp^R^	Novagen
pCDFDuet-1	CDF ori with P_T7_; Sm^R^	Novagen
pET-A	pETDuet-1 carrying *aibA*, *aibB*, *aibC* and *liuC*	This study
pCDF-E	pCDFDuet-1 carrying *ERG10* and *ERG13*	This study
pCDF-EV1	pCDFDuet-1 carrying *ERG10*, *ERG13* and *HlVPS* ^*syn*^	This study
pCDF-EV2	pCDFDuet-1 carrying *ERG10*, *ERG13* and *FvCHS2*-*1* ^*syn*^	This study
pCDF-EV3	pCDFDuet-1 carrying *ERG10*, *ERG13* and *HpCHS*	This study
Strains		
Myxobacteria	*Myxococcus xanthus* (CGMCC 1.3865)	CGMCC
Yeast	*Saccharomyces cerevisiae* BY4742	29
APG-0	*E. coli* BL21 (DE3) with pETDuet-1 and pCDFDuet-1	This study
APG-IV	*E. coli* BL21 (DE3) with pET-A and pCDF-E	This study
APG-1	*E. coli* BL21 (DE3) with pET-A and pCDF-EV1	This study
APG-2	*E. coli* BL21 (DE3) with pET-A and pCDF-EV2	This study
APG-3	*E. coli* BL21 (DE3) with pET-A and pCDF-EV3	This study

*CGMCC* China general microbiological culture collection center

### Construction of plasmids

Oligonucleotide primers used in this study are summarized in Additional file 1: Table S1. *AibA*, *aibB*, *aibC* and *liuC* were PCR-amplified from *Myxococcus xanthus* (CGMCC 1.3865) genome with primer pairs aibA-F/aibA-R, aibB-F/aibB-R, aibC-F/aibC-R or liuC-F/liuC-R, respectively. The amplified gene products of *aibA*, *aibB* and *aibC* were digested with restriction enzyme and simultaneously subcloned into the multiple cloning sites (MCS) 1 of pETDuet-1 by Golden Gate [25] cloning using *Aar*I. Subsequently, the gene product of *liuC* was digested and ligated into the above resulting plasmid using *Hin*dIII/*Afl*II to yield the final plasmid pET-A. Here, the sequences of T7 promoter, lac operator and ribosome binding site (RBS) from pETDuet-1 were incorporated into the primer design, so each gene was driven by a single T7 promoter. *ERG10* and *ERG13* were PCR-amplified from *Saccharomyces cerevisiae* BY4742 genome with primer pairs ERG10-F/ERG10-R or ERG13-F/ERG13-R, respectively. First, the *ERG13* fragment was digested and cloned into *Nco*I/*BamH*I sites of pCDFDuet-1. The *ERG10* fragment was digested and ligated into the above plasmid via *Nde*I/*Xho*I sites, generating the recombinant plasmid pCDF-E. The *HlVPS* gene from hops (GenBank: AB015430.1) was codon optimized for expression in *E. coli* and synthesized by Shanghai Generay Biotech Co., Ltd. (Shanghai, China), designated as *HlVPS*^*syn*^ (Additional file 1: Table S2). *HlVPS*^*syn*^ was PCR-amplified with primer pair VPS-F/VPS-R, and cloned into pET28a through *Nde*I/*Afl*II sites generating the intermediate expression vector pET28a-HlVPS. The *T7*-*HlVPS*^*syn*^ fragment with a T7 promoter and an N-terminal His tag was PCR-amplified from pET28a-HlVPS with primer pair T7VPS-F/T7VPS-R, digested and ligated into pCDF-E via *Not*I/*Afl*II to generate the final recombinant plasmid pCDF-EV1. *FvCHS2*-*1* from strawberry (GenBank: XM_004306495.1) was codon optimized for expression in *E. coli* and synthesized by Shanghai Generay Biotech Co., Ltd. (Shanghai, China), designated as *FvCHS2*-*1*^*syn*^ (Additional file 1: Table S2). *HpCHS* was obtained from the mRNA of *H. perforatum* by RT-PCR. RNA of *H. perforatum* callus was isolated by RNAprep pure Plant Kit from Tiangen Biotech Co., Ltd. (Beijing, China). Reverse transcription was performed using the First Strand cDNA Synthesis Kit from Toyobo Co., Ltd. (Osaka, Japan). PCR reactions were performed using High Fidelity PCR system (Fermentas, Germany) with primer pair HpCHS-F/HpCHS-R. The *HpCHS* sequence was submitted into NCBI database (GenBank: KU180217). *FvCHS2*-*1*^*syn*^ and *HpCHS* were ligated into pCDF-E individually using the same method as pCDF-EV1 construction, yielding pCDF-EV2 and pCDF-EV3, respectively.

### Biosynthesis of isovaleryl-CoA in *E. coli*

The plasmids, pET-A and pCDF-E, were electroporated into BL21 (DE3), generating the recombinant strain APG-IV. The strain APG-0 harboring pETDuet-1 and pCDFDuet-1 was used as the control. A 1 mL aliquot of the overnight cultured single colony was inoculated into 50 mL LB medium containing antibiotics and cultivated at 37 °C, 200 rpm with shaking. When OD_600_ of the culture reached 0.6, 0.1 mM isopropyl-β-D-thiogalactoside (IPTG) was added to induce recombinant protein expression at 30 °C for 12 h. Subsequently, the cells were centrifuged (4000 rpm, 30 °C, 10 min), washed and resuspended in 50 mL modified M9 medium and cultured at 30 °C for 3 h. The cell culture was chilled on ice for 10 min. Cells were harvested by centrifugation (4000 rpm, 4 °C, 10 min) and resuspended in 1.5 mL extraction buffer (methanol:acetonitrile:water = 2:2:1, v/v, with 0.1 % formic acid). After sonication and centrifugation, 50 μL of the supernatants were used for HPLC and LC–MS analysis.

### Biosynthesis of PIVP and HIBP in *E. coli*

*Escherichia coli* strain BL21 (DE3) was transformed with plasmids pET-A and pCDF-EV1, pET-A and pCDF-EV2 or pET-A and pCDF-EV3 to generate the recombinant strains APG-1, APG-2 or APG-3, respectively. Two stage fermentation processes were applied to achieve the production of PIVP and HIBP. The first stage is for pathway genes overexpression, and recombinant strains were cultivated in TB medium. Single colonies were cultured in 3 mL LB medium with antibiotics at 37 °C for 7 h as seed culture, 1 mL of which was transferred into 50 mL TB medium with antibiotics and cultured in a shake flask at 37 °C until an OD_600_ of 1.2 followed by addition of 0.1 mM IPTG. To determine optimal temperature for fermentation conditions at the stage, the culture was incubated at 16, 23 or 30 °C for 16 h. Different concentrations of IPTG (0.01, 0.05, 0.1, 0.2 or 0.3 mM) were also added in the TB medium to determine the optimal concentrations. The cell pellets were harvested by centrifugation and resuspended in an equal volume of modified M9 medium and fermented at 30 °C, 200 rpm for secondary metabolite production as the second stage. Time course for production of PIVP and HIBP by strain APG-1 was determined for 12, 24, 36, 48 and 60 h. Subsequently, the culture broth was centrifuged at 4000 rpm for 10 min. The supernatants were extracted with an equal volume of ethyl acetate twice, concentrated by rotary evaporator, and re-dissolved in 1.5 mL methanol. To extract the intracellular metabolites, cell pellets were resuspended in 10 mL 80 % acetone, sonicated, centrifuged and the supernatant was evaporated to remove the acetone. The procedure was repeated once. Subsequently, the remaining water phase was also extracted with an equal volume of ethyl acetate twice and re-dissolved in 1.5 mL methanol as the supernatant of the fermented broth. Then 20 µL extraction samples of the fermentation broth or the cell pellets were analyzed by HPLC–MS separately.

### Compounds purification

2 L fermentation broth of the recombinant strain APG-1 was centrifuged to separate into supernatant and cell pellets. After crude extraction using the above protocol, purification of HIBP and PIVP was conducted by semi-preparative HPLC performed on a Shimadzu LC-6 AD with SPD-20A detector. The HPLC conditions were as follows: solvent A = H_2_O; solvent B = methanol; flow rate: 4 mL/min, 0–5 min 90 % A and 10 % B, 6–40 min 90 % A and 10–100 % B (linear gradient), 41–45 min 100 % B. A YMC-pack ODS-A (10 × 250 mm; particle size, 5 μm) was used as the stationary phase.

### Chemical analysis and quantification

HPLC–MS was performed on an Agilent 1260 system with 1260 Infinity UV detector and a Bruker microQ-TOF II mass spectrometer equipped with an ESI ionization probe. The innoval C18 column (4.6 × 250 mm; 5 μm particle size) was used in this study. The HPLC conditions for isovaleryl-CoA, HIBP and PIVP were as follows: solvent A = H_2_O (containing 20 mM ammonium acetate for the analysis of isovaleryl-CoA, or containing 0.1 % formic acid for the analysis of HIBP and PIVP); solvent B = methanol; flow rate = 1 mL/min; 0–5 min 95 % A and 5 % B, 6–45 min 95 % A and 5 % B to 100 % B (linear gradient). All these products were detected at 254 nm. Standard calibration curve of isovaleryl-CoA was generated with a series of known concentrations of the isovaleryl-CoA standard purchased from Sigma-Aldrich (Milwaukee, WI, USA). Standard calibration curves of HIBP and PIVP were generated with a series of known concentrations of the purified compounds from large scale fermentation. All experiments were carried out in triplicate and repeated at least twice. The titer was presented as mean ± SD.

### NMR analysis

NMR experiments were performed on a Bruker Avance 400 (Karlsruhe, Germany). The sample were dried by evaporation and dissolved in 500 µL of DMSO-*d*_6_ and transferred into 2.5 mm NMR tube. Chemical shifts were expressed in *δ* (ppm) and coupling constants (*J*) were given in Hertz (Hz) (Additional file 1: Figure S2).

*HIBP*^1^H-NMR (DMSO-*d*_6_, 400 MHz) *δ* 5.94 (d, *J* = 2.1 Hz, 1H), 5.21 (d,* J* = 2.1 Hz, 1H), 2.30 (d, *J* = 7.2 Hz, 2H), 1.93 (m, 1H), 0.89 (d, *J* = 6.6 Hz, 6H);

*PIVP*^1^H-NMR (DMSO-*d*_6_, 400 MHz), *δ* 5.79 (s, 2H), 2.86 (d, *J* = 6.8 Hz, 2H), 2.13 (m, 1H), 0.91 (d, *J* = 6.7 Hz, 6H).

## Results

### Engineered synthesis of isovaleryl-CoA in *E. coli*

**Fig. 3:**
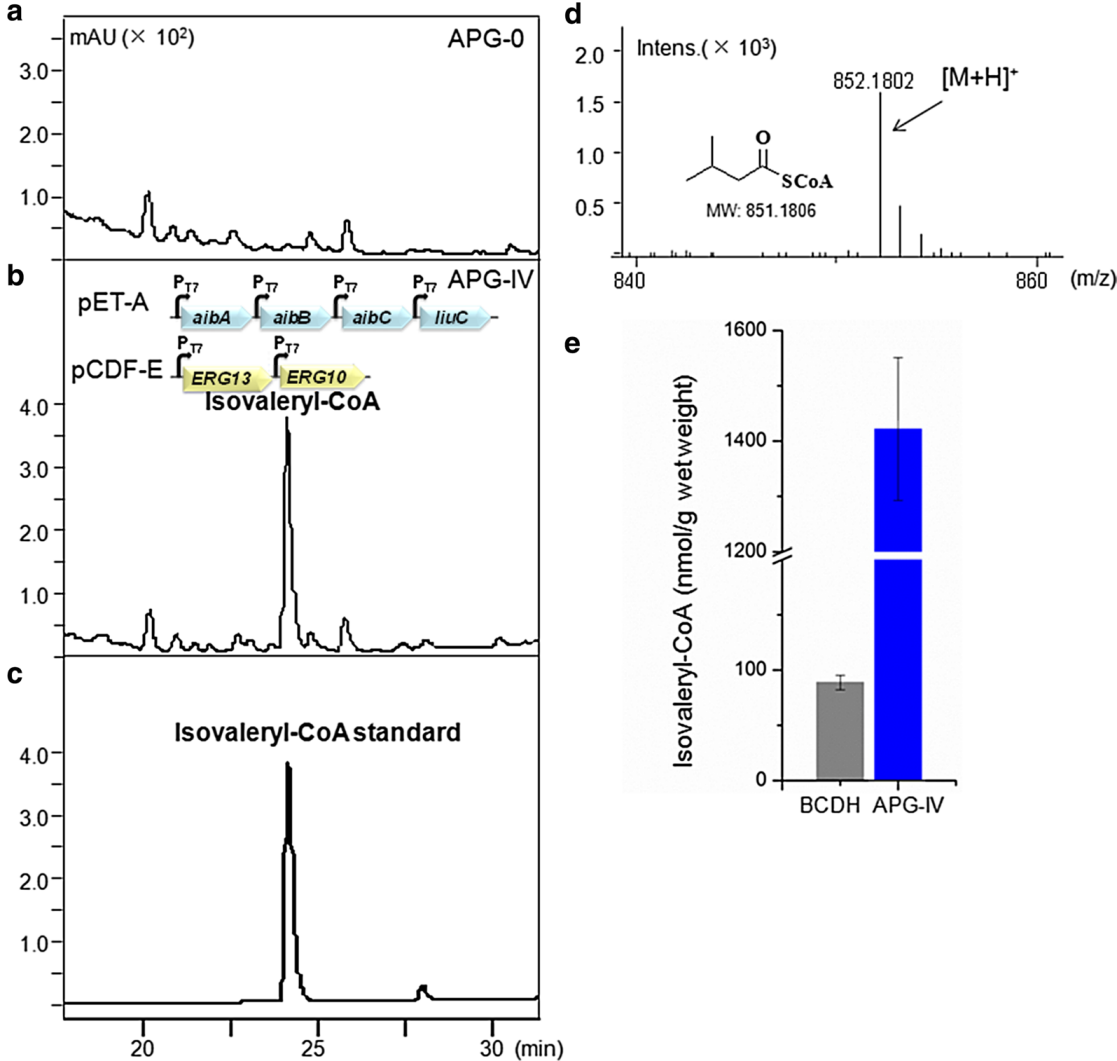
Engineered synthesis of isovaleryl-CoA by *E. coli* strains. **a** HPLC analysis of chemical profiles produced by *E. coli* strain APG-0 harboring empty vectors. **b** HPLC analysis of isovaleryl-CoA produced by *E. coli* strain APG-IV carrying pET-A and pCDF-E. **c** The isovaleryl-CoA standard. **d** MS chromatogram showing the synthesis of isovaleryl-CoA. **e** Titers of isovaleryl-CoA produced by the recombinant strain APG-IV and our previous *E. coli* expressing the BCDH complex [26]

First, we generated isovaleryl-CoA in *E. coli* cells employing a pathway from *M. xanthus* via hydroxy-3-methylglutaryl CoA (HMG-CoA), a key intermediate of mevalonate pathway in the biosynthesis of isoprenoids [24]. Short branched-chain acyl-CoAs are usually derived from the branched-chain amino acids as a mixture via transamination and subsequent oxidative decarboxylation catalyzed by the branched-chain α-keto acid dehydrogenase complex (BCDH) [26]. In current work, acetyl-CoA is converted into HMG-CoA as in the mevalonate pathway (Fig. 2a). Enzymes ERG10 (acetyl-CoA acetyltransferase) and ERG13 (HMG-CoA synthase) from *S. cerevisiae* were used for efficient biosynthesis of HMG-CoA, as these two enzymes yielded enhanced levels of mevalonate in *E. coli* [27–29]. The three enzymes from *M. xanthus*, including LiuC (HMG-CoA dehydratase), AibA/B (3-methylglutaconyl-CoA (MG-CoA) decarboxylase) and AibC (3,3-dimethylacrylyl CoA (DMA-CoA) reductase), were used to transform HMG-CoA into isovaleryl-CoA consecutively via MG-CoA and DMA-CoA in the above *E. coli* strain (Fig. 2a). Acyl-CoAs were extracted from the engineered *E. coli* strain APG-IV harboring pET-A and pCDF-E and analyzed by HPLC–MS with strain APG-0 harboring pETDuet-1 and pCDFDuet-1 as the negative control (Fig. 3). This procedure confirmed the production of isovaleryl-CoA by comparing the retention time (t_*R*_ = 24.5 min) and the molecular ion [(M + H)^+^ = 852.1802] with those of the isovaleryl-CoA standard (Fig. 3b, c). The titer of isovaleryl-CoA from this new pathway was 1421.8 ± 129.2 nmol/g wet weight, about sixteen times higher than the titer of our previous engineered *E. coli* strain using the BCDH complex under the same fermentation and detection conditions (Fig. 3d) [26].

### Engineered synthesis of PIVP and HIBP in *E. coli* carrying *HlVPS*^*syn*^ and optimization of fermentation conditions

**Fig. 4:**
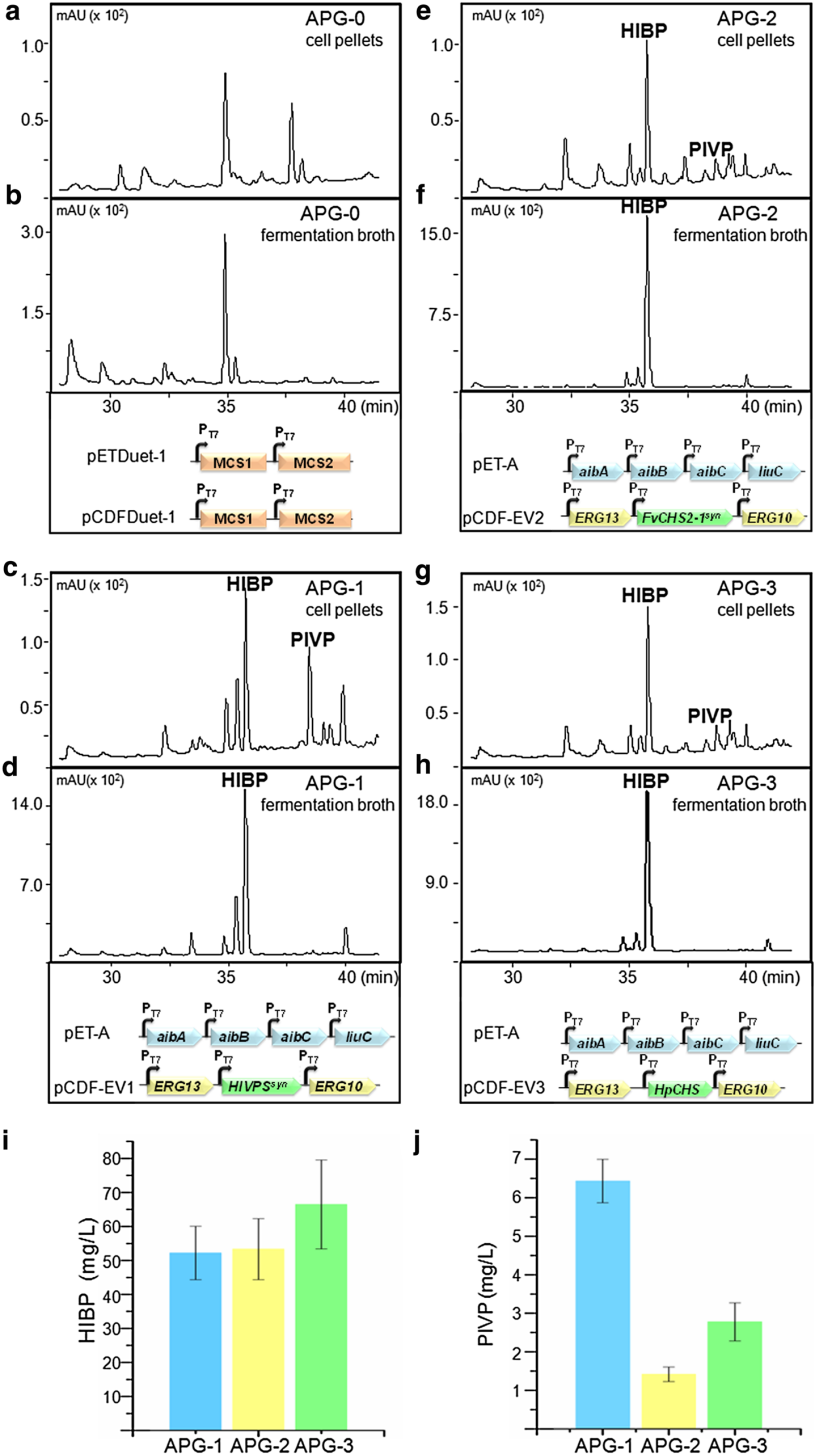
Engineered synthesis of 4-hydroxy-6-isovaleryl-2-pyrone (HIBP) and phlorisovalerophenone (PIVP) by *E. coli* strains. **a**, **b** Strain APG-0 harboring empty vectors as negative control. **c**, **d** Strain APG-1 harboring plasmids pET-A and pCDF-EV1. **e**, **f** Strain APG-2 harboring plasmids pET-A and pCDF-EV2. **g**, **h** Strain APG-3 harboring plasmids pET-A and pCDF-EV3. **a**, **c**, **e**, **g** HPLC analysis of metabolites extracted from cell pellets. **b**, **d**, **f**, **h** HPLC analysis of metabolites extracted from the fermentation broth. **i** The titers of HIBP produced by the strains APG-1, APG-2 and APG-3. **j** The titers of PIVP produced by the strains APG-1, APG-2 and APG-3

The *HlVPS*^*syn*^ from hops was synthesized and introduced into the isovaleryl-CoA producing *E. coli* strain, generating the recombinant strain APG-1. After fermentation, the broth supernatants and cell pellets were analyzed by HPLC–MS. As shown in the chromatogram, the recombinant strain APG-1 produced two new compounds (Fig. 4c, d), compared with the control strain APG-0 (Fig. 4a, b). The first compound with a t_*R*_ of 35.6 min was initially identified as HIBP by LC–MS (Additional file 1: Figure S2a, c; [M + H]^+^ = 169.0763). In the previous enzymatic studies, the production of HIBP was confirmed only by LC–MS analysis probably due to no enough products for NMR analysis [3, 23]. In this work, about 74.9 mg HIBP was isolated from 2 L scale culture, and we were able to confirm the structure by 1D-NMR spectroscopy analysis (Additional file 1: Figure S2a, c). The second product with a t_*R*_ of 39.7 min was identified as PIVP (Additional file 1: Figure S2b, [M + H]^+^ = 211.0925). About 6.5 mg HIBP was isolated from 2 L culture, and the PIVP structure was further confirmed by ^1^H-NMR spectroscopy analysis (Additional file 1: Figure S2d). The localization of the two products was also tested. PIVP mainly accumulated in the cell pellets (Fig. 4c), while HIBP was distributed in the fermentation broth as well as the cell pellets (Fig. 4c, d).

**Fig. 5:**
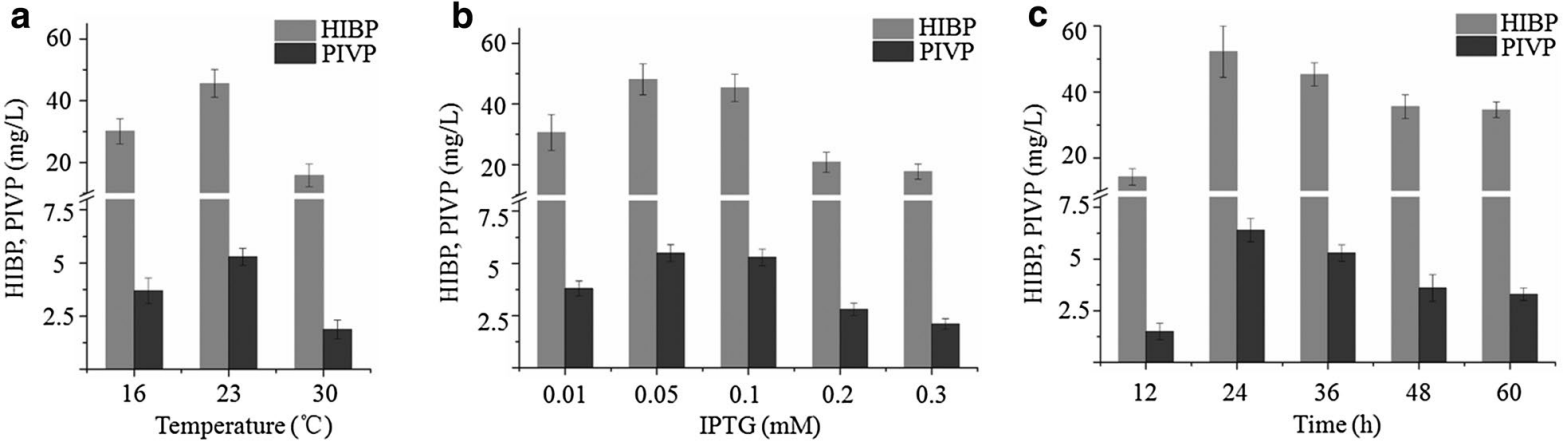
Optimization of fermentation conditions. **a** The titers of PIVP and HIBP by strain APG-1 at different protein induction temperature. **b** The titers of PIVP and HIBP by strain APG-1 at different IPTG concentration. **c** The titers of PIVP and HIBP by strain APG-1 at different fermentation time

We further optimized the production of PIVP and HIBP by varying fermentation conditions for the recombinant strain APG-1 such as temperature and IPTG concentration at the first stage. The recombinant strain APG-1 was incubated in TB medium for 16 h at varying temperature (16, 23 or 30 °C) with a fixed IPTG concentration of 0.1 mM. The cell pellets were harvested and resuspended in modified M9 medium and incubated at 30 °C for 36 h. Combining both the fermentation broth and cell pellets, the titerss of PIVP and HIBP exhibited best at 23 °C (Fig. 5a). After that, different concentrations of IPTG, 0.01, 0.05, 0.1, 0.2 and 0.4 mM were used to induce protein expression at 23 °C. The titers of PIVP and HIBP were highest when the concentration of IPTG was 0.05 mM as shown in Fig. 5b. Furthermore, we monitored titers of PIVP and HIBP produced by APG-1 cultivated in modified M9 medium at different fermentation time. The results are summarized in Fig. 5c. The titers of HIBP and PIVP reached the highest in 24 h, which were 52.0 ± 7.9 and 6.4 ± 0.6 mg/L, respectively. Under these concentrations, no inhibition on cell growth by the compounds HIBP and PIVP was observed.

### Biosynthesis of HIBP and PIVP in *E. coli* using CHSs

In addition to HlVPS, a CHS from strawberry (*F. vesca*), FvCHS2-1 played a role as a bifunctional CHS/VPS enzyme [3]. We performed codon optimization for the *FvCHS2*-1 gene, synthesized and introduced the *FvCHS2*-1^syn^ into the isovaleryl-CoA producing *E. coli* strain, generating APG-2 and fermented under the optimized conditions. The protein expression was induced at 23 °C for 16 h with 0.05 mM IPTG, and then the fermentation was conducted in modified M9 medium at 30 °C for 24 h. HPLC analysis showed that the strain APG-2 also produced HIBP and PIVP, although the titer of PIVP was much lower than that carrying *HlVPS*^*syn*^, only 1.4 ± 0.2 mg/L (Fig. 4e, f, j). However, the titer of HIBP reached 53.3 ± 9.0 mg/L, which was similar to that of the strain harboring *HlVPS*^*syn*^ (Fig. 4i).

VPS was involved in the formation of PIVP in the biosynthesis of humulone and could also produce PIPB using isobutyryl-CoA as the starter unit [21–23]. PIBP is the core structure of hyperforin, an antidepressant extracted from *Hypericum* species. The enzyme catalyzing formation of PIBP is still unknown. It was assumed that there may be a VPS-like or dual functional CHS/VPS enzyme in the *Hypericum* species responsible for the formation of PIBP, and more than likely, this enzyme could also use isovaleryl-CoA as the starter molecular to form PIVP and HIBP. Based on the reported type III PKSs gene sequences of the *Hypericum* species [30] in the NCBI database, several candidate genes were amplified from the callus of *H. perforatum* by homology cloning. Among these genes, a suspected CHS gene showing 96.2 % similarity with FvCHS2-1 at the amino acid level was designated as *HpCHS* (GenBank: KU180217), and introduced into the above isovaleryl-CoA producer. The recombinant *E. coli* strain APG-3 harboring the isovaleryl-CoA pathway genes and *HpCHS* was fermented as described above. The HIBP titer produced by this strain reached 66.5 ± 13.1 mg/L, which was the highest among all the three producers (Fig. 4g–i). The PIVP titer in APG-3 was 2.8 ± 0.5 mg/L, which was lower than that of the APG-1 harboring *HlVPS*^*syn*^ (Fig. 4g, h, j).

## Discussion

Short branched-chain acyl-CoAs are important building blocks for a large number of valuable compounds [5, 24, 31]. However, native *E. coli* metabolism does not produce these short branched-chain acyl-CoAs, which hampers the heterologous production of those economically important chemicals in this host. Previously engineered short branched-chain acyl-CoA producing *E. coli* strains usually biosynthesized isobutyryl-CoA, isovaleryl-CoA and 2-methyl-butyryl-CoA as a mixture derived from the metabolic pathways of branched-chain amino acids catalyzed by the BCDH complex [26, 32, 33]. In this study, we successfully demonstrated the feasibility of synthesizing isovaleryl-CoA in *E. coli* via HMG-CoA (Fig. 2a) recruiting five enzymes from yeast and myxobacteria [24]. Isovaleryl-CoA was the only product in this pathway which makes the engineered *E. coli* strain more suitable for producing compounds such as humulone and lupulone with isovaleryl-CoA as the building block. In the BCDH dependent pathway, isovaleryl-CoA and isobutyryl-CoA are derived from α-ketoisocaproate or α-ketoisovalerate directly, and α-ketoisovalerate is an intermediate for the formation of α-ketoisocaproate. Thus the formation of isobutyryl-CoA competes with isovaleryl-CoA in the pathway (Fig. 2a). Meanwhile short branched-chain keto acids would also be transformed into corresponding amino acids such as leucine, isoleucine and valine beyond acyl-CoAs (Fig. 2a). HMG-CoA formation with ERG10 and ERG13 derived from acetyl-CoA has been well established in the mevalonate pathway (Fig. 2a) in *E. coli* [27–29]. By recruiting LiuC, AibA/B and AibC, the HMG-CoA was further uniquely diverted into the formation of isovaleryl-CoA via MG-CoA and DMA-CoA. This engineered strain represents a novel “factory” for biosynthesis of a series of valuable chemicals with isovaleryl-CoA as the building block, including fatty acids, biofuels and natural products [26, 32, 33].

Introduction of VPS from hops, CHSs from strawberry or *H. perforatum* to the above isovaleryl-CoA producing *E. coli* strain resulted in the biosynthesis of PIVP and HIBP. PIVP was detected in hop extracts [34], and is the key intermediate of humulone, which contribute the characteristic bitter flavor of beer and display multiple pharmacological effects [4, 31]. The work described here may pave the way for synthesis of humulone and other derivatives. HIBP has not been isolated from any natural resources [11–13]. As an analogue of TAL, HIBP could be used as a potential platform chemical to produce various chemical intermediates and end products with short branched-chains (Additional file 1: Figure S1a, b) [11–13, 35]. More experiments are needed to test whether HIBP itself has any antibacterial or fungicidal activity or not.

In this study, the VPS from hops was the best enzyme for PIVP formation. The newly cloned CHS from *H. perforatum* was the most appropriate enzyme for the synthesis of HIBP. The ratios of HIBP and PIVP were calculated based on the titers produced by strains APG-1 (52.0 over 6.4 mg/L), APG-2 (53.3 over 1.4 mg/L) and APG-3 (66.5 over 2.8 mg/L), which were about 8:1, 37:1 and 24:1, respectively. In the in vitro enzyme activity analysis of HlVPS and FvCHS, PIVP was the major product, instead of HIBP [3, 22]. It was reported that the PIVP and HIBP content was affected by the relative ratio of isovaleryl-CoA/malonyl-CoA in the in vitro experiment [3]. In our recombinant *E. coli*, the production of isovaleryl-CoA and malonyl-CoA both derived from acetyl-CoA, and there may be a dynamic balance between the concentration of isovaleryl-CoA and malonyl-CoA in vivo (Fig. 2a), which may affect the ratio of HIBP and PIVP.

Amino acids sequence alignment of HlVPS, FvCHS2-1 and HpCHS showed high similarity (Additional file 1: Figure S4). The three essential catalytic amino acids Cys-His-Asn, the active site loop of CHS enzymes GFGPG, and two Phe residues, important in determining the substrate specificity of CHS are all well conserved in these three proteins (Additional file 1: Figure S4). However, ratios between PIVP and HIBP produced by these three enzymes are quite different (Fig. 4). Several protein engineering studies have elucidated the basis of starter molecule selectivity and the control of polyketide length of type III PKSs. For example, Jez and colleagues reported that a triple mutant (T197L/G256L/S338I) of CHS, generated an enzyme that was functionally identical to 2-PS, catalyzing the synthesis of the TAL from an acetyl-CoA starter molecule and two malonyl-CoAs [36]. In 2005, Abe and co-workers reported that a point mutation (M207G) in a pentaketide chromone synthase (PCS) expanded the volume of the catalytic cavity, to convert PCS into octaketide synthase [37]. Thus, modeling analyses and site-directed mutagenesis experiments are needed to improve the product specificity and increase the synthesis of PIVP or HIBP.

Directed evolution may be also used to increase the activity of HlVPS, FvCHS2-1 or HpCHS. Using directed evolution, Rao and colleagues obtained a series of thermostable PhlD mutants with improved phloroglucinol productivity [19]. Recently, Tang and colleagues engineered the *E. coli* regulatory protein AraC to activate gene expression in response to TAL, developed an endogenous TAL reporter system in *E. coli.* Using this system, they conducted in vivo directed evolution of 2-PS in *E. coli,* and obtained a 2-PS variant conferring ~20-fold higher TAL production [6]. Similar reporter systems may also be constructed in our recombinant *E. coli* to facilitate high throughput screening of HlVPS, FvCHS2-1 or HpCHS for improved productivity of HIBP or PIVP. At the same time, precursor supply including isovaleryl-CoA and malonyl-CoA may be improved for the production of HIBP and PIVP. The availability of intracellular malonyl-CoA may be enhanced by over-expressing the acetyl-CoA carboxylase (Acc) and acetyl-CoA synthase (Acs) genes and deletion of competing pathways, which was applied in improving phloroglucinol productivity by engineered *E. coli* strain [19, 38]. In the case of HIBP and PIVP, as both substrates isovaleryl-CoA and malonyl-CoA were derived from acetyl-CoA, there may be a dynamic balance between these two substrates and may need more elaborate regulation.

## Conclusions

In this work, we firstly constructed a biosynthetic pathway of isovaleryl-CoA in *E. coli* by recruiting a route via hydroxy-3-methylglutaryl CoA, and then investigated the potential of bio-renewable production of PIVP and HIBP with *E. coli* as the host using type III PKSs. Even though still in infancy, the work described here may pave the way for microbial synthesis of not only acylphloroglucinols derivatives, but also “unnatural” TAL analogues.

## Additional file

10.1186/s12934-016-0549-9 The main primers used in this study. **Table S2.** DNA sequences of synthesized genes. **Figure S1.** TAL was demonstrated to be a platform chemical and HIBP may also be used as a potential platform chemical. **Figure S2.** Mass spectrum and ^1^H-NMR analysis of HIBP and PIVP. **Figure S3.** Strain APG-IV and the *E. coli* strain harboring pET28a-HlVPS were also used as negative controls for the production of HIBP/PIVP. **Figure S4.** Comparison of the deduced amino acid sequences of HpCHS, FvCHS2-1 and HlVPS.

## Abbreviations

2-PS: 2-pyrone synthase
BCDH: branched-chain α-keto acid dehydrogenase complex
CHS: chalcone synthase
DMA-CoA: 3,3-dimethylacrylyl CoA
HIBP: 4-hydroxy-6-isobutyl-2-pyrone
HIPP: 4-hydroxy-6-isopropyl-2-pyrone
HMG-CoA: hydroxy-3-methylglutaryl CoA
IPTG: isopropyl-β-d-thiogalactoside
MG-CoA: 3-methylglutaconyl-CoA
PCS: pentaketide chromone synthase
PIBP: phlorisobutyrophenone
PIVP: phlorisovalerophenone
PKSs: polyketide synthases
TAL: triacetic acid lactone
VPS: valerophenone synthase
