# Supplementary material for: Biosynthesis of phlorisovalerophenone and 4-hydroxy-6-isobutyl-2-pyrone in Escherichia coli from glucose
Source: Microb Cell Fact. 2016 Aug 30;15(1):149. doi: 10.1186/s12934-016-0549-9 (PMC5004256; doi:10.1186/s12934-016-0549-9)
Supplement: Supplementary file 1 — 10.1186/s12934-016-0549-9 The main primers used in this study. Table S2. DNA sequences of synthesized genes. Figure S1. TAL was demonstrated to be a platform chemical and HIBP may also be used as a potential platform chemical. Figure S2. Mass spectrum and 1H-NMR analysis of HIBP and PIVP. Figure S3. Strain APG-IV and the E. coli strain harboring pET28a-HlVPS were also used as negative controls for the production of HIBP/PIVP. Figure S4. Comparison of the deduced amino acid sequences of HpCHS, FvCHS2-1 and HlVPS. [file 12934_2016_549_MOESM1_ESM.docx]

**Biosynthesis of** **phlorisovalerophenone and 4-hydroxy-6-isobutyl-2-pyrone in *Escherichia coli* from glucose**

Wei Zhou^1,2,3^, Yibin Zhuang^1,2^, Yanfen Bai^1,2,3^, Huiping Bi^1,2,*^, Tao Liu^1,2,*^, and Yanhe Ma^1^

1 Tianjin Institute of Industrial Biotechnology, Chinese Academy of Sciences, Tianjin 300308, China

2 Key Laboratory of Systems Microbial Biotechnology, Chinese Academy of Sciences, Tianjin 300308, China

3 University of Chinese Academy of Sciences, Beijing, China

Address correspondence to Huiping Bi, bi_hp@tib.cas.cn; Tao Liu, liu_t@tib.cas.cn

**Supplementary Table 1** The main primers used in this study

| **Primers** | **Oligonucleotide sequences (5'-3')** | **Restriction site** |
| --- | --- | --- |
| ERG13-F | CATTTTCCATGGTGAAACTCTCAACTAAACTTTGTTG | *Nco*I |
| ERG13-R | CATTTTGGATCCTTATTTTTTAACATCGTAAGATC | *Bam*HI |
| ERG10-F | cattttcatatgtctcagaacgtttacattg | *Nde*I |
| ERG10-R | CATTTTCTCGAGTCATATCTTTTCAATGACAATAG | *Xho*I |
| pETduet-F | CACACCACACCTGCAAAAttcccctgtagaaataattttgtttaactttaataaggagatataagcttgcggccgcataatgcttaag | *Aar*I |
| pETduet-R | CACACCACACCTGCAAAAATGGTATATCTCCTTCTTAAAG | *Aar*I |
| aibA-F | CACACCACACCTGCAAAAccatgaagacggcgcgctggtg | *Aar*I |
| aibA-R | CACACCACACCTGCAAAAACAGGGGAATTGTTATCCGCTCACAATTCCCCTATAGTGAGTCGTATTATCATGCCGCCCTCCGCGTCAG | *Aar*I |
| aibB-F | CACACCACACCTGCAAAActgtagaaataattttgtttaactttaataaggagatataccatgagcgcgacgctggacatcac | *Aar*I |
| aibB-R | CACACCACACCTGCAAAACAGGGGAATTGTTATCCGCTCACAATTCCCCTATAGTGAGTCGTATTATCAGGCGCCGACGAGCGCGTC | *Aar*I |
| aibC-F | CACACCACACCTGCAAAAcctgtagaaataattttgtttaactttaataaggagatataccatgaaagccgtcgtactgcgcag | *Aar*I |
| aibC-R | CACACCACACCTGCAAAAGGAATTGTTATCCGCTCACAATTCCCCTATAGTGAGTCGTATTATCACGCCTCGGGGGGCACCAG | *Aar*I |
| liuC-F | cattttaagcttatgccggaattcaaggtcgac | *Hin*dIII |
| liuC-R | cattttCTTAAGCTAGCGGCCCTTGTAGACGG | *Afl*II |
| VPS-F | aaaaGATATACATGGCCAGCGTGACAGTGGAGC | *Nde*I |
| VPS-R | AAAActtaagttacacgtttgtaggcacgctgt | *Afl*II |
| T7VPS-F | aaaagcggccgccgatcccgcgaaattaatacgac | *Not*I |
| T7VPS-R | AAAActtaagttacacgtttgtaggcacgctgt | *Afl*II |
| HpCHS-F | aaaacatatggtgaccgtggaagaagtcaggaagg | *Nde*I |
| HpCHS-R | AAAAGGATCCTTAATATGCGACACTGTGAAGGACCAC | *Bam*HI |
| T7HpCHS-F | aaaagcggccgccgatcccgcgaaattaatacgac | *Not*I |
| T7HpCHS-R | AAAActtaagTTAATATGCGACACTGTGAAGG | *Afl*II |
| T7FvCHS-F | aaaagcggccgccgatcccgcgaaattaatacgac | *Not*I |
| T7FvCHS-R | AAAActtaagTTAGGCTGCAACGCTATGTAACA | *Afl*II |

**Supplementary Table 2** DNA sequences of synthesized genes

| **Gene** | **DNA Sequence** |
| --- | --- |
| *HlVPS^syn^* | ATGGCCAGCGTGACAGTGGAGCAGATTCGTAAGGCACAGCGTGCAGAGGGCCCTGCCACCATTCTGGCAATTGGCACCGCCGTTCCGGCCAACTGCTTCAACCAGGCCGATTTCCCTGACTACTACTTTCGCGTGACCAAGAGTGAGCACATGACCGACCTGAAGAAGAAGTTCCAGCGCATGTGCGAGAAGAGCACCATCAAAAAGCGCTACCTGCACCTGACAGAAGAGCACCTGAAGCAGAACCCGCACTTATGCGAGTACAACGCCCCGAGTCTGAATACCCGCCAAGACATGCTGGTGGTTGAAGTGCCGAAGCTGGGCAAGGAAGCCGCAATCAACGCCATCAAGGAATGGGGTCAGCCGAAGAGTAAGATCACCCACCTGATCTTCTGCACCGGCAGCAGTATTGATATGCCGGGCGCCGACTATCAGTGCGCCAAGCTGTTAGGCCTGCGCCCGAGTGTGAAGCGCGTTATGCTGTACCAGCTGGGCTGTTACGCAGGCGGCAAAGTGCTGCGCATCGCCAAGGACATCGCCGAGAATAACAAGGGCGCACGCGTGTTAATCGTGTGCAGCGAGATCACCGCATGTATTTTCCGTGGTCCGAGCGAGAAGCATCTGGATTGCCTGGTGGGCCAGAGCCTGTTCGGCGATGGTGCCAGCAGTGTTATCGTTGGCGCAGACCCTGACGCCAGTGTGGGTGAACGCCCGATCTTCGAGTTAGTGAGTGCAGCCCAGACCATCTTACCGAATAGCGATGGCGCCATCGCCGGTCACGTTACCGAAGCAGGTCTGACCTTCCACTTACTGCGTGACGTTCCGGGCCTGATCAGCCAGAACATCGAGAAGAGCCTGATCGAGGCCTTCACCCCGATCGGCATTAACGACTGGAACAACATCTTCTGGATTGCCCACCCGGGTGGTCCTGCAATCCTGGACGAGATCGAGGCCAAGCTGGAGCTGAAGAAGGAGAAGATGAAAGCCAGCCGTGAGATGCTGAGCGAGTACGGCAATATGAGTTGCGCCAGTGTGTTCTTCATCGTGGACGAGATGCGCAAACAGAGCAGCAAAGAAGGCAAGAGCACCACCGGTGATGGCCTGGAATGGGGTGCCCTGTTCGGTTTTGGCCCGGGCTTAACCGTGGAGACCGTTGTGTTACACAGCGTGCCTACAAACGTGTAA |
| *FvCHS2-1^syn^* | ATGGTGACCGTGGAAGAAGTTCGTAAAGCACAGCGCGCAGAAGGTCCGGCAACCGTGCTGGCCATTGGGACAGCCACCCCGCCGAATTGTATTGATCAGTCTACCTATCCGGATTATTATTTTCGCATTACCAATAGCGAACATAAAGCAGAACTGAAAGAAAAATTTCAGCGCATGTGTGATAAATCTATGATTAAAAAACGCTATATGTATCTGACCGAAGAAATTCTGAAAGAAAATCCGAGTATGTGTGAATATATGGCCCCGTCTCTGGATGCACGCCAGGATATGGTTGTGGTTGAAATTCCGAAACTGGGTAAAGAAGCCGCAGTTAAAGCAATTAAAGAATGGGGTCAGCCGAAATCTAAAATTACCCACTTAGTGTTTTGTACCACCTCAGGCGTTGATATGCCGGGTGCCGATTATCAGTTAACCAAACTGTTAGGCTTACGCCCGAGCGTTAAACGTTTAATGATGTATCAGCAGGGTTGTTTTGCAGGCGGCACCGTGCTGCGCTTAGCCAAGGATCTGGCAGAAAACAACCGCGGTGCACGGGTGTTAGTTGTGTGTAGCGAAATTACCGCAGTGACCTTTCGCGGTCCGAGCGATACCCATCTCGATAGCTTAGTGGGCCAGGCCCTGTTTGGCGATGGCGCCGCAGCAATTATTGTGGGTAGTGATCCGCTGCCGGAAGTGGAACGTCCGCTGTTTGAACTGGTGAGTGCCGCACAGACCATTCTGCCGGATTCAGATGGTGCCATTGATGGTCATCTGCGCGAAGTGGGCTTAACCTTTCATCTGCTGAAAGATGTTCCGGGCCTGATTTCTAAAAATATTGAAAAATCACTGAACGAAGCCTTTAAACCGCTGAATATTACCGATTGGAATAGCTTATTTTGGATTGCACATCCGGGCGGTCCGGCAATTTTAGATCAGGTGGAAGCCAAACTGGCCCTGAAACCGGAAAAACTGGAAGCAACCCGTCATATTCTGTCAGAATATGGTAATATGTCTTCAGCATGTGTGCTGTTTATTTTAGATGAAGTTCGTCGTCGCAGTGCAGCCAATGGTCATAAAACCACCGGCGAAGGCAAAGAATGGGGCGTTCTGTTTGGCTTTGGACCTGGCCTGACCGTGGAAACCGTTGTGTTACATAGCGTTGCAGCCTAA |

**
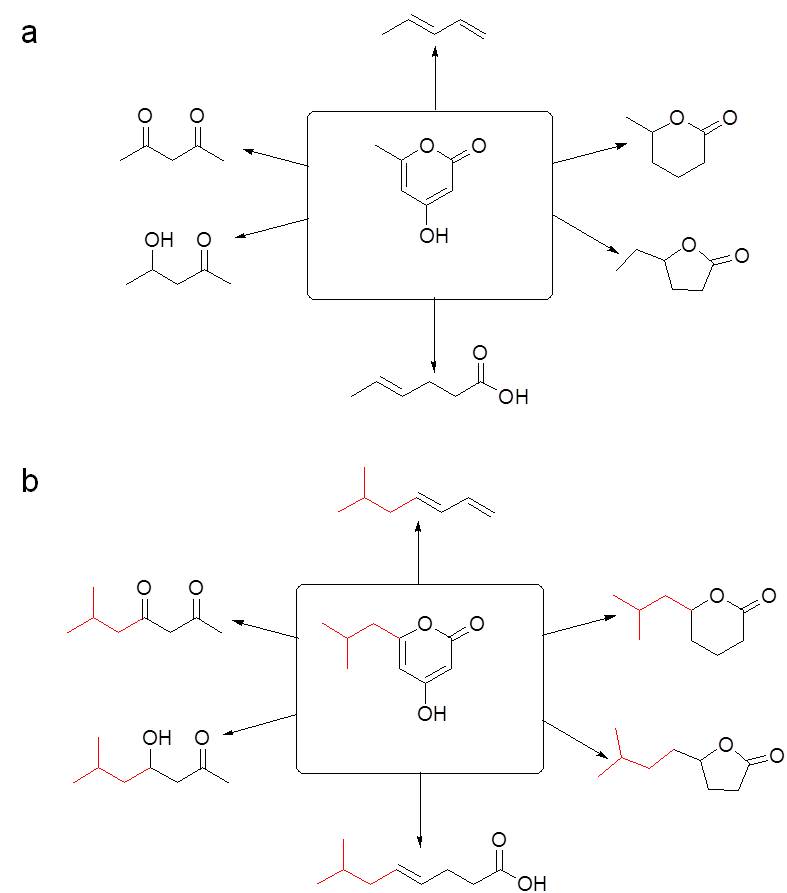
**

**Figure S1.** (a) Triacetic acid lactone (TAL) was demonstrated to be a platform chemical for the production of commercially valuable chemical intermediates and end products, such as 2,4-pentanedione/acetylacetone, 4-hydroxy-2-pentanone, hexenoic acid, δ-hexalactone, γ-caprolactone and 1,3-pentadiene [1]. (b) As an analogue of TAL, 4-hydroxy-6-isobutyl-2-pyrone (HIBP) may also be used as a potential platform chemical for the production of chemical intermediates and end products with short-branched chain [2].


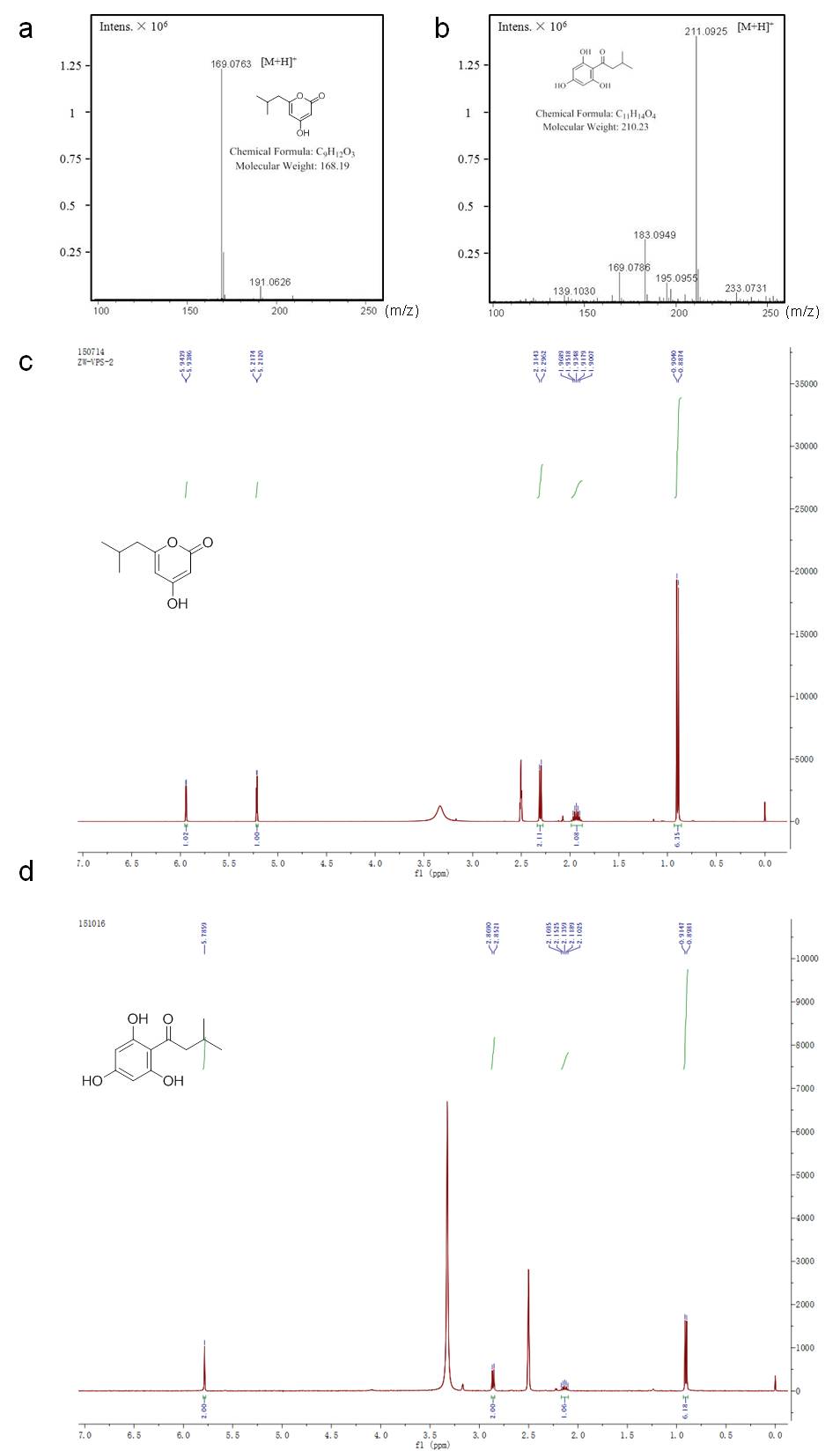


**Figure S2.** (a) Mass spectrum of 4-hydroxy-6-isovaleryl-2-pyrone (HIBP). (b) Mass spectrum of phlorisovalerophenone (PIVP). (c) ^1^H-NMR analysis of HIBP. (D) ^1^H-NMR analysis of PIVP.


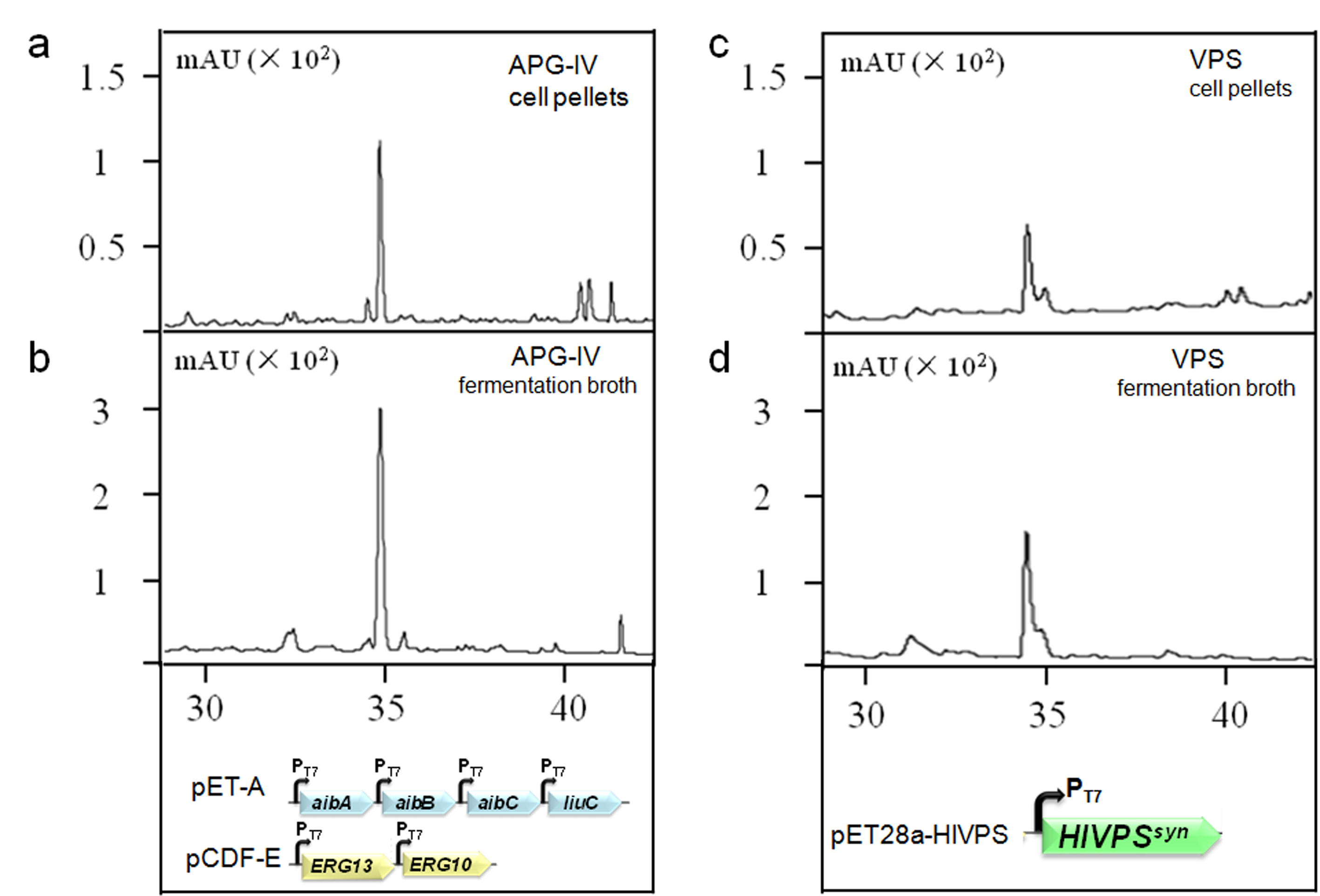


**Figure S3**. Strain APG-IV and the *E. coli* strain harboring pET28a-*HlVPS^syn^* were also used as negative controls for the production of HIBP/PIVP. (a) and (b) Strain APG-IV harboring the isovaleryl-CoA producing pathway but without VPS/CHS was analyzed as a negative control for the production of HIBP/PIVP. (c) and (d) *E. coli* strain harboring pET28a-HlVPS without a complete pathway for production of isovaleryl-CoA was analyzed as another negative control for the production of HIBP/PIVP.

**Figure S4**. Comparison of the deduced amino acid sequences of HpCHS, FvCHS2-1 and HlVPS. The sequences were aligned using the ClustalW program. The conserved catalytic triad Cys-His-Asn is boxed in green. The highly conserved active site loop of CHS enzymes, GFGPG is boxed in yellow and two Phe residues, important in determining the substrate specificity of CHS are boxed in red.

**References**

1. Chia M, Schwartz TJ, Shanks BH, Dumesic JA: **Triacetic acid lactone as a potential biorenewable platform chemical.** *Green Chemistry* 2012, **14:**1850-1853.

2. Dumesic JA, Chia M: **Production of 2, 4-diones from 4-hydroxy-6-substituted-2-pyrones.** Google Patents; 2013, US 20120283477A1.
